# Refining Cardiovascular Calcification in the Appraisal of Ischemic Heart

**DOI:** 10.7759/cureus.64328

**Published:** 2024-07-11

**Authors:** Vinh Nguyen, Jerry Fan, Imran Hafeez, Antoine Nguyen, Hamid Mojibian

**Affiliations:** 1 Cardiology, Baylor Scott and White Medical Center - Temple, Temple, USA; 2 Radiology, Yale School of Medicine, Yale University, New Haven, USA; 3 Internal Medicine, University of North Texas Health Science Center, Fort Worth, USA

**Keywords:** coronary artery disease, single photon emission tomography (spect), coronary computed tomography angiogram, coronary artery calcification, ischemic heart diseases

## Abstract

Background: Even in asymptomatic patients, there is a high association of ischemia on myocardial perfusion scans in those with coronary artery calcification or valvular calcifications. Patients without coronary artery calcifications have exceeding-low rates of cardiovascular events. The absence of cardiovascular calcification, including coronary artery, valvular, and thoracic aorta is a strong negative predictor of myocardial ischemia. In individuals with suspected ischemia who had chest computed tomography imaging, evaluation for cardiovascular calcification (coronary artery, valves, and thoracic aorta) is an invaluable tool to guide management for further diagnostic imaging. We hypothesize that the complete absence of cardiovascular calcification has a high negative predictive value for defects in myocardial perfusion imaging such as single-photon emission computed tomography (SPECT) or positron emission tomography (PET).

Methods: Non-contrast computed tomography performed for SPECT/PET CT attenuation correction from March 1, 2017, to September 30, 2017, were retrospectively reviewed for the absence of cardiovascular calcification by a cardiologist and radiologist who were blinded to patients' medical history. Medical records were reviewed to include patient demographics and medical history. A total of 132 patients were analyzed.

Results: Of the 132 patients without calcifications, seven patients had small myocardial perfusion defects suggestive of ischemia or infarct, but none were considered significant defects. Of these seven patients, six were managed medically and one was from an outside institution with no follow-up data. Two of the seven patients had follow-up invasive angiography or coronary CTA that did not show significant atherosclerotic coronary artery disease.

Conclusion: A complete absence of cardiovascular calcification indicates a 100% negative predictive value for a significant perfusion defect on same-day confirmatory nuclear stress testing. Patients with suspected ischemia but absent cardiovascular calcifications can be safely managed medically without further testing for ischemia.

## Introduction

The evolution of computed tomography (CT) has ushered in an era of cost-effective and efficient evaluation of coronary artery disease (CAD) [1]. Coronary artery calcification (CAC) identified in CT is a reliable predictor of adverse cardiovascular events and is a well-subscribed indicator of coronary atherosclerotic plaque burden [1]. Conversely, the absence of coronary artery calcification is an excellent negative predictive value of significant coronary artery disease (less than 2% prevalence of obstructive CAD, defined as >50% stenosis) on invasive angiography, low incidence of myocardial ischemia, and overall good prognosis [1]. In addition, the absence of CAC in both symptomatic and asymptomatic patients has a low probability of significant stenosis, abnormal myocardial perfusion test, and low likelihood of coronary artery disease. Therefore, downstream testing may not be needed in this specific population [2,3]. CAC is often combined with CT coronary angiography (CTCA), which can be a valuable tool to assess non-calcified lesions, grade stenosis, and evaluate for anomalous coronary arteries that can cause sudden cardiac death [4].

Myocardial perfusion scan has often been used to assess ischemia, the presence of calcifications in the coronary arteries (prevalence of ischemia of 22%), and aorta/valves (prevalence of ischemia 36%) are highly associated with positive findings on myocardial perfusion scans due to the correlation of calcification with inflammation, lipoprotein deposits, and endothelial dysfunction [5,6]. The additive role of absent calcification of the valves and thoracic aorta on top of CAC has not been thoroughly investigated. In this study, we assessed non-contrast chest CT performed for single-photon computed tomography (SPECT) or positron computed tomography (PET) attenuation correction for complete absence of coronary artery, valvular, and thoracic aorta calcification. We aimed to determine the prevalence of perfusion defects in such patients, the utility of myocardial perfusion imaging (MPI) in this scenario, and the clinical outcome. Given the low likelihood of ischemia in studies with absent calcifications, the role of a non-dedicated CT scan (those that are not classified as a computed tomography angiography {CTA} coronary or CT calcium score, and used for a different purpose) can lower the resource utilization of downstream testing and also help clinicians make a critical decision about further testing for ischemia [5,6].

## Materials and methods

**Figure 1:**
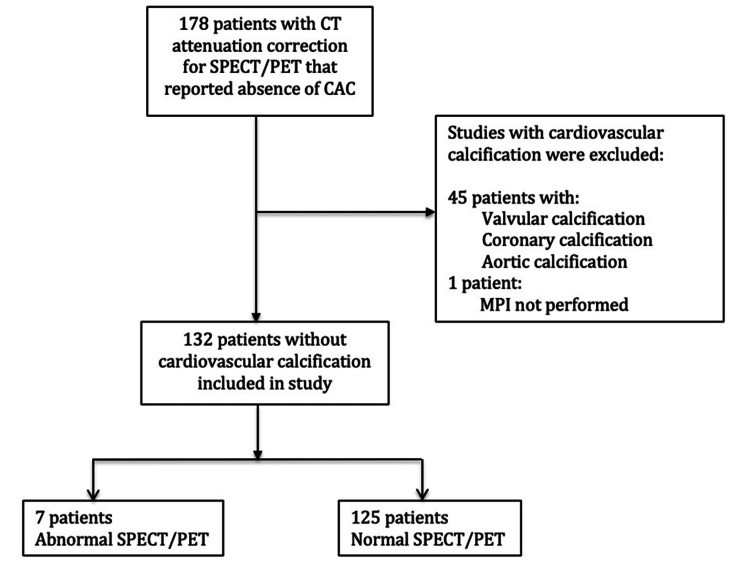
Patients with the absence of cardiovascular calcification were dichotomized based on abnormal or normal SPECT/PET results. SPECT: single-photon emission computed tomography; PET: positron emission tomography; CAC: coronary artery calcification; MPI: myocardial perfusion imaging

We retrospectively searched MONTAGE (mPower by Burlington, MA: Nuance Communications, Inc.) to identify adult patients (>18 years old) who had a non-contrast CT performed for attenuation correction (120 kVp, non-gated) as part of MPI using SPECT or PET at Yale New Haven Hospital from March 2017 through September 2017 [7]. The referral pool was predominantly outpatient (96%). Our standard CT report notes the presence or absence of CAC. We filtered studies that noted absence of CAC. A total of 178 patients were identified. CT images were then reviewed by a cardiac radiologist and cardiologist to confirm the absence of cardiac calcification. We further excluded studies with valvular and/or thoracic aorta calcification (Figure 1). A total of 132 patients who did not have any cardiovascular calcifications met the inclusion criteria. Those with perfusion defects on PET/SPECT were correlated with invasive angiography or coronary CTA (if performed). Additional patient data including demographics, comorbidities, and cardiovascular medications were obtained from the electronic medical record.

## Results

A total of 132 patients without cardiovascular calcification were eligible for the study. Each study was reviewed by a board-certified cardiologist or radiologist trained in reading CT scans and myocardial perfusion scans. The mean age was 52.9 years. There were 22% of patients with diabetes, 22% with hyperlipidemia, 41% with hypertension, 42% with obesity, and 20% with a history of tobacco smoking. Medications included 33% angiotensin-converting enzyme inhibitor, 27% beta blocker, and 38% statin therapy (Table 1). The overall comorbidities and medications for our patient population are lower than other similar studies [5].

**Table 1:** Baseline characteristics, comorbidities, and medications. ACE-I: angiotensin-converting enzyme inhibitors; ARB: angiotensin II receptor blockers; n: number of patients; PAD: peripheral artery disease

Characteristics	Value, n (%)
Average age (years)	52.9
Female	54 (42%)
Outpatient	125 (96%)
Comorbidities	
Chronic kidney disease	4 (3%)
Diabetes	27 (21%)
Hyperlipidemia	28 (22%)
Hypertension	52 (40%)
Obesity	54 (42%)
PAD	1 (<1%)
Tobacco smoking	26 (20%)
Medications	
ACE-I/ARB	43 (33%)
Beta-blocker	35 (27%)
Calcium channel antagonist	15 (12%)
Mineralocorticoid receptor antagonist	2 (2%)
Statin therapy	49 (38%)

Regarding SPECT/PET perfusion, seven of 132 patients had findings indicating ischemia or infarct. Areas of perfusion defects were small, mild in severity indicating a score of 1 or 2 on perfusion (Table 2). Of these seven patients, two underwent follow-up invasive angiography or coronary CTA that did not show significant coronary artery disease. The negative predictive value of absence of cardiovascular calcification for significant perfusion defect (defined as greater than mild, which is a perfusion defect severity of 1 or 2, graded from 0-4, mild=1 or 2, moderate=2, severe=3, absent=4) was 100% [8].

**Table 2:** Patients with abnormal MPI, follow-up invasive angiography or coronary CTA if performed, and clinical outcome. CAD: coronary artery disease; CTA: computed tomography angiography; MPI: myocardial perfusion imaging; NICM: non-ischemic cardiomyopathy; RCA: right coronary artery; LAD: left anterior descending artery; LVEF: left ventricular ejection fraction; RA: right atrium

Patient	Indication	Perfusion findings	Follow-up angiogram	Clinical course
1	Chest pain, dyspnea	Small, mild apical inferolateral ischemia	No evidence of atherosclerotic CAD	Medical management
2	Chest pain	Small, mild mid to apical anterior ischemia	No evidence of atherosclerotic CAD. Coronary-cameral fistula of the RCA to the RA, minimal mid-LAD bridging	Medical management
3	Preoperative, non-cardiac surgery	Small, mild mid to apical inferior and inferoseptal ischemia	None	Underwent surgery without complications
4	Dyspnea	Small, mild apical inferior infarct	Unknown	Outside referral. No follow-up data
5	Chest pain	Small, very mild basal to apical inferolateral ischemia	None	Symptoms spontaneously resolved
6	Chest pain	Small, mild apical anterior infarct	None	Medical management
7	Chest pain, decreased LVEF	Small, mild mid to apical anteroseptal ischemia	None	Medical management of NICM

## Discussion

Coronary artery calcium is a powerful tool for risk stratification and prognosis [1]. A CAC 0 was associated with an annual event rate of 0.3% over a 5.2-year follow-up [1]. In a large study of 3,914 consecutive patients, the absence of coronary artery calcium has an excellent negative predictive value of 99.5% for obstructive coronary artery disease and this effectively rules out the risk of coronary events during a 13-year follow-up period [1]. Thus, CAC can be a cost-effective method for excluding significant coronary artery disease in low-to-intermediate risk patients with stable symptoms and safely followed in the outpatient setting without the need for additional costly diagnostic imaging studies for five years (only 2% progressed to a CAC >50) [2]. Additionally, even greater confidence is achieved with zero CAC and the absence of valvular and thoracic aorta calcification.

Our study analyzed patients with not only the absence of CAC but also an absence of valvular and thoracic aorta calcifications. Of the 132 patients, 5% (seven patients) had perfusion defects. Our findings are similar to a prior meta-analysis that showed 4,870 patients who underwent MPI and CAC scoring, with the absence of CAC associated with 6% ischemia and a negative predictive value of 99% for ruling out acute coronary syndrome [2]. In our cohort, given the high negative predictive value for significant perfusion defects, an argument can be made to omit the stress perfusion portion and consider non-cardiac causes of chest pain. In a large study of 3,895 patients who underwent CACS, a subset of the population (411 patients) also underwent SPECT MPI, no subjects with CACS <10 had ischemia on SPECT MPI, compared to 2.6% with CACS 11-100, 11.3% with CACS 101-399, and 46% with CACS 400 [3]. If continued suspicion of cardiac chest pain persists, a coronary CTA may be considered instead of a perfusion study to assess myocardial bridging, coronary vasculitis, or anomalous coronary origins, particularly when myocardial perfusion imaging is insufficient to diagnose these conditions and may yield false-positive or false-negative results [4]. Realize that coronary CTA, compared to functional imaging in stable chest pain, does not lead to a difference in primary outcomes but can result in higher rates of cardiac catheterization (12.2% vs 8.1%), according to the Prospective Multicenter Imaging Study for Evaluation of Chest Pain (PROMISE) trial [9]. This may be explained by the excellent sensitivity of coronary CTA to detect disease (99%) at the expense of lower specificity (64%) due to the overestimation of severity of stenosis, with a positive predictive value of 64% [10]. However, the application of non-invasive fractional flow reserve derived from CT has shown superior diagnostic accuracy and discrimination of flow-limiting lesions [4].

Since our studied population was predominantly outpatient (96%) who were low-intermediate risk based on traditional atherosclerotic cardiovascular disease (ASCVD) criteria, the data should be interpreted with caution if extrapolated to the higher-risk cohort. In the Coronary Artery Evaluation Using 64-Row Multidetector Computed Tomography Angiography (CORE 64) trial, high-risk symptomatic patients (at high risk for coronary artery disease) had a 20% incidence (14 out of 72) of at least one angiographic stenosis at least 50% despite having no CAC on computed tomography [11]. Despite being a smaller study, it is important to underscore that CAC is reserved for low-to-intermediate-risk patients in accordance with Bayes' theorem [11].

In an effort to reduce the cost of needing dedicated computed tomography imaging for patients referred for chest pain evaluation, in many instances there are prior chest imaging studies that can help assess calcification burden and help assess the risk of coronary artery disease. As a cost-saving measure, assessing for cardiovascular calcification on prior chest CT for other indications can help guide management. Although CAC on non-gated chest CT is not scored in a standardized manner, its presence is associated with increased cardiovascular risk [1,2,10,12]. A prior report showed that the incidence of acute coronary syndrome in low-to-intermediate risk patients was 1.1% (2/183) in those without CAC compared to 31% (77/248) in those with CAC [2]. Identification of CAC can alter primary prevention in a way perfusion imaging cannot, and leads to appropriate and greater use of statin and antiplatelet therapy [13].

The study has several limitations, including a relatively small sample size and retrospective single-center study, which limits the generalizability of the findings of this study. In addition, our study population when compared to our study has fewer comorbid conditions and is on less medical therapy. In addition, due to the utilization of a non-contrasted CT, we are not detecting soft plaque which is not calcified and would not show up in these studies. Despite the limited number of patients in our study at a single center, our findings resonate prior larger-scale investigations, but in a real-life uncontrolled setting.

## Conclusions

The correlation between calcification and coronary artery disease has previously been established. We believe that adding valvular and thoracic aorta calcification on top of coronary artery calcification adds greater value to the prognostication of cardiovascular disease burden. The presence of cardiovascular calcification is not a measure of current disease but a barometer of future events. By the same token, in the absence of cardiovascular calcification, efforts should be diverted away from ischemia evaluation in low-to-intermediate-risk patients which reduces the healthcare burden. Further prospective studies are needed to validate the data in our pilot study. Given the plurality of CT scans that are acquired in the ED to “rule out” other conditions can be an excellent tool to help clinicians assess cardiovascular risk.

## References

[1] Mittal TK, Pottle A, Nicol E (2017). Prevalence of obstructive coronary artery disease and prognosis in patients with stable symptoms and a zero-coronary calcium score. Eur Heart J Cardiovasc Imaging.

[2] Sarwar A, Shaw LJ, Shapiro MD (2009). Diagnostic and prognostic value of absence of coronary artery calcification. JACC Cardiovasc Imaging.

[3] He ZX, Hedrick TD, Pratt CM, Verani MS, Aquino V, Roberts R, Mahmarian JJ (2000). Severity of coronary artery calcification by electron beam computed tomography predicts silent myocardial ischemia. Circulation.

[4] Cheezum MK, Liberthson RR, Shah NR, Villines TC, O'Gara PT, Landzberg MJ, Blankstein R (2017). Anomalous aortic origin of a coronary artery from the inappropriate sinus of valsalva. J Am Coll Cardiol.

[5] Yamazato R, Yamamoto H, Tadehara F (2012). Association between aortic valve calcification and myocardial ischemia, especially in asymptomatic patients. J Nucl Med.

[6] Fathala A, Al Amer A, Shukri M, Abouzied MM, Alsugair A (2012). The relationship between coronary artery calcification and myocardial perfusion in asymptomatic women. Ann Saudi Med.

[7] mPower Clinical Analytics for medical imaging. https://www.nuance.com/healthcare/diagnostics-solutions/radiology-performance-analytics/mpower-clinical-analytics.html.

[8] Dvorak RA, Brown RK, Corbett JR (2011). Interpretation of SPECT/CT myocardial perfusion images: common artifacts and quality control techniques. Radiographics.

[9] Douglas PS, Hoffmann U, Patel MR (2015). Outcomes of anatomical versus functional testing for coronary artery disease. N Engl J Med.

[10] Meijboom WB, Meijs MF, Schuijf JD (2008). Diagnostic accuracy of 64-slice computed tomography coronary angiography: a prospective, multicenter, multivendor study. J Am Coll Cardiol.

[11] Gottlieb I, Miller JM, Arbab-Zadeh A (2010). The absence of coronary calcification does not exclude obstructive coronary artery disease or the need for revascularization in patients referred for conventional coronary angiography. J Am Coll Cardiol.

[12] Greenland P, Bonow RO, Brundage BH (2007). ACCF/AHA 2007 clinical expert consensus document on coronary artery calcium scoring by computed tomography in global cardiovascular risk assessment and in evaluation of patients with chest pain: a report of the American College of Cardiology Foundation Clinical Expert Consensus Task Force (ACCF/AHA writing committee to update the 2000 expert consensus document on electron beam computed tomography) developed in collaboration with the Society of Atherosclerosis Imaging and Prevention and the Society of Cardiovascular Computed Tomography. J Am Coll Cardiol.

[13] Hulten E, Bittencourt MS, Singh A (2014). Coronary artery disease detected by coronary computed tomographic angiography is associated with intensification of preventive medical therapy and lower low-density lipoprotein cholesterol. Circ Cardiovasc Imaging.

